# Dietary supplementation with n-3-fatty acids in patients with pancreatic cancer and cachexia: marine phospholipids versus fish oil - a randomized controlled double-blind trial

**DOI:** 10.1186/s12944-017-0495-5

**Published:** 2017-06-02

**Authors:** Kristin Werner, Daniela Küllenberg de Gaudry, Lenka A. Taylor, Tobias Keck, Clemens Unger, Ulrich T. Hopt, Ulrich Massing

**Affiliations:** 1 Institute of Surgical Pathology, Medical Center-University of Freiburg, Freiburg, Germany; 2 German Cochrane Center Freiburg, Medical Center-University of Freiburg, Freiburg, Germany; 3 Department of Surgery, Medical Center-University of Freiburg, Freiburg, Germany; 4 Pharmacy, Ruprecht-Karls-University Heidelberg, Heidelberg, Germany; 5 Clinic for Surgery, Medical Center University of Lübeck, Lübeck, Germany; 6 Cancer Center Freiburg, Freiburg, Germany; 7 Institute of Pharmaceutical Science, University of Freiburg, Freiburg, Germany; 8 Tumor Biology Center Freiburg, Freiburg, Germany

**Keywords:** Pancreatic cancer, Cancer cachexia, Marine phospholipids, Fish oil, Medium chain triglycerides, N-3-fatty acids, Weight stabilization, Randomized controlled trial

## Abstract

**Background:**

Like many other cancer patients, most pancreatic carcinoma patients suffer from severe weight loss. As shown in numerous studies with fish oil (FO) supplementation, a minimum daily intake of 1.5 g n-3-fatty acids (n-3-FA) contributes to weight stabilization and improvement of quality of life (QoL) of cancer patients. Given n-3-FA not as triglycerides (FO), but mainly bound to marine phospholipids (MPL), weight stabilization and improvement of QoL has already been seen at much lower doses of n-3-FA (0,3 g), and MPL were much better tolerated. The objective of this double-blind randomized controlled trial was to compare low dose MPL and FO formulations, which had the same n-3-FA amount and composition, on weight and appetite stabilization, global health enhancement (QoL), and plasma FA-profiles in patients suffering from pancreatic cancer.

**Methods:**

Sixty pancreatic cancer patients were included into the study and randomized to take either FO- or MPL supplementation. Patients were treated with 0.3 g of n-3-fatty acids per day over six weeks. Since the n-3-FA content of FO is usually higher than that of MPL, FO was diluted with 40% of medium chain triglycerides (MCT) to achieve the same capsule size in both intervention groups and therefore assure blinding. Routine blood parameters, lipid profiles, body weight, and appetite were measured before and after intervention. Patient compliance was assessed through a patient diary. Quality of life and nutritional habits were assessed with validated questionnaires (EORTC-QLQ-C30, PAN26). Thirty one patients finalized the study protocol and were analyzed (per-protocol-analysis).

**Results:**

Intervention with low dose n-3-FAs, either as FO or MPL supplementation, resulted in similar and promising weight and appetite stabilization in pancreatic cancer patients. MPL capsules were slightly better tolerated and showed fewer side effects, when compared to FO supplementation.

**Conclusion:**

The similar effects between both interventions were unexpected but reliable, since the MPL and FO formulations caused identical increases of n-3-FAs in plasma lipids of included patients after supplementation. The effects of FO with very low n-3-FA content might be explained by the addition of MCT. The results of this study suggest the need for further investigations of marine phospholipids for the improvement of QoL of cancer patients, optionally in combination with MCT.

## Background

Cancer may induce severe weight loss (cancer cachexia), which is a common problem of pancreatic cancer patients. It is defined as a continuous and unintended weight loss of at least 5% since first diagnosis over a period of six months [1]. Approximately 80% of all patients with advanced pancreatic cancer die because of cancer cachexia [1]. This condition is usually accompanied with appetite loss and lower quality of life (QoL) [2]. Various studies have shown that patients who achieved weight gain during cancer cachexia had lower mortality rates [3]. Several reasons for cancer cachexia have been reported. One of the most important reasons is the tumor associated inflammatory response, which is a multifactorially induced metabolic disorder. The activity of pro-inflammatory mediators like the cytokines TNFα, IL-1 and IL-6 as well as INF-γ lead to catabolic processes like lipolysis, proteolysis, and increased resting energy expenditure [2, 4–6] The cytokine release stimulates the production of pro-inflammatory eicosanoids like PGE_2_, PGF_2a_, PGD_2_, PGI_2_ and TXA_2_ – all bioactive lipid-second-messengers [7, 8] derived from arachidonic acid (AA), which is a n-6-polyunsaturated fatty acid (20:4 (n − 6)).

AA is an important component of cellular membranes, where it is predominantly bound to the 2-position of membrane forming phospholipids. Especially in inflammatory events, AA is cleaved from the phospholipids and serves at least in part for pro-inflammatory eicosanoid production and hence contributes to the development of cancer cachexia.

If the pro-inflammatory AA (a n-6-FA) are partly replaced by the anti-inflammatory n-3-polyunsaturated fatty acids docosahexaenoic acid (DHA) and eicosapentaenoic acid (EPA), i.e. by consuming higher amounts of DHA and EPA, then the n-3-FAs will compete with AA for biosynthesis of lipid-second-messengers. In contrast to AA, metabolic transformation of DHA and EPA results in the production of anti-inflammatory eicosanoids like prostaglandins of the first and third class and leukotrienes of the fifth class. This displacement of AA in the phospholipid component of cellular membranes by EPA or DHA results in reduced inflammatory response induced by pro-inflammatory cytokines and may therefore reduce cancer cachexia [9–12]

The anti-inflammatory effects of oral n-3-FA – mostly given as fish oil (FO) - have broadly been investigated. Barber has shown that EPA, given as nutritional supplement, increased weight of 20 pancreatic carcinoma patients [13], and a similar effect has been shown by Wigmore et al. [14] In a meta-analysis, Colomer et al. [12] summarized some high quality studies focusing on n-3-FA supplementation in cancer patients suffering from weight loss. This meta-analysis demonstrated a weight, appetite, and life quality stabilizing effect of n-3-FA when giving a minimum dose of 1.5 g per day over a period of at least eight weeks. Newer interventional studies have confirmed effects of n-3-FAs on weight stabilization and on the reduction of pro-inflammatory cytokines [15–19]. However, there are controversial results. For example, Bruera et al. [20] found no significant weight improvement in 60 cachectic cancer patients taking EPA compared to placebo. Similarly, Fearon et al. [21, 22] failed to demonstrate a weight stabilizing effect of EPA in two studies (2.2 g and 2 g/4 g of EPA, respectively) supplementation, compared to placebo or protein and energy dense supplements in cachectic cancer patients. A common problem of these studies was also the low compliance of patients to FO, since the application ofFO is often accompanied by gastrointestinal side effects like “fishy” regurgitation and taste, nausea or flatulence if the dose exceeded the minimal effective dose of 1.5 g n-3 FA daily [23, 24]. To avoid these side effects and to get better effects on body weight-, appetite- and QoL, current studies investigated n-3-FA given as marine phospholipids (MPL) [8] (and not as FO), which mainly consist of n-3-FA-carrying phospholipids. In the GI-tract, MPL are rapidly dispersed into the GI-fluids (as nanoparticles), which is in contrast to FO which form larger oil-droplets. In addition to the avoidance of large FO droplets mainly localized on top of the gastric juice, nanodispersed MPL will be much faster digested. This explains (i) the observed better effects of MPL compared to FO, and (ii) the reduced GI side effects. For this reason, patient’s compliance to phospholipid bound n-3-FA is supposed to be better than for FO formulation.

Furthermore, several studies confirmed a higher integration of EPA and DHA into the cellular membranes, when these FAs were given as phospholipids instead of triglycerides [25, 26]. In this regard, studies of Burri et al. [27], Schuchardt et al. [28], Ramprasath et al. [29] and Ulven et al. [30] demonstrated a rapid supply and high bioavailability of the n-3-FA bound to phospholipids (interventional studies). In a four-week study with 24 healthy volunteers, Ramprasath et al. compared n-3-FA-carrying phospholipids (krill oil) containing 600 mg of EPA and DHA with FO and a placebo. They found a higher n-3-FA uptake into the blood plasma, when compared to n-3-FA as FO and to placebo (corn oil) [29]. A few years earlier, Taylor et al. [8] investigated n-3-FA-carrying phospholipids (marine phospholipids from salmon roe) on 17 cachectic cancer patients over six weeks. They found weight stabilization, and an improvement of appetite as well as QoL even at a very low dose of FAs (corresponding to 300 mg EPA and DHA per day), while the compliance was high. Additionally, inflammation – measured as CRP – decreased significantly duringthe intervention.

Since the study of Taylor was performed with patients suffering from different cancer types and was a non-controlled interventional trial, the aim of the present study was to compare the effect of an identical composition (ratio of DHA and EPA) and low amount of n-3-FA (300 mg/day) either given as MPL- and as FO-formulation on body weight, appetite global health (QoL) and on the FA-profiles in plasma in pancreatic cancer patients. Both interventions provided an amount of 300 mg EPA and DHA daily, and were given either as MPL of as FO.

## Methods

### Study design and participants

We undertook a randomized, double-blind, controlled trial in the International Pancreatic Cancer Center Freiburg, in the Department of Oncology of the Medical Center University of Freiburg, and the Freiburg Tumor Biology Center between February 2011 and February 2011. Inclusion criteria were the following: pancreatic carcinoma, minimal age of 18 years, life expectancy of at least three months, tumor associated weight loss of at least 5% since diagnosis, Karnofsky score of at least 60%, no allergy against fish or seafood, oral nutrition, no blood coagulation disorders and no psychological disorder. Sixty patients were assessed for eligibility and allocated for intervention. They were randomly assigned to receive either MPL or FO. Randomization and masking was performed with assignment envelopes containing the letter A or B, which were prepared by a non-involved external party (Membramed GmbH). Assignment code was revealed at the end of the study by Membramed GmbH.

All of the participants gave informed written consent. The study protocol was approved by the Ethics Committee of the University of Freiburg, Germany (Ethics Committee number 25/10, study number DRKS0000345, UTN U1111–1113-6181).

### Study protocol

Patients were asked to take one capsule (MPL or FO) three times a day along with meals for six weeks. The FO and MPL supplement consisted of similar amounts of n-3-fatty acids (DHA and EPA) in the same ratio, and was offered as 500 mg soft gel capsules. FO capsules contained 60% of FO and 40% of medium chain triglycerides (MCT) (6.9 g/100 g eicosapentaenoic acid (EPA) and 13.6 g/100 g docosahexaenoic acid (DHA)). MPL capsules contained 35% of n-3-FA-phospholipids (mainly phosphatidylcholine) plus 65% of neutral lipids (8,5 g/100 g EPA and 12.3 g/100 g DHA). The final n-3-FA dose was 300 mg/day in both groups. Both capsules were provided by Membramed AG, Hamburg, Germany. Concerning appearance, taste and smell, there was no difference between the two preparations. Assignment to each preparation was done in a double-blind manner (FO: Capsules A; MPL: Capsules B).

The primary endpoint was the change of weight and appetite. Secondary endpoints included QoL, fatty acid profile of blood plasma, nutritional status, routine blood parameters and adherence.

Each patient received a patient diary, in which daily body weight, appetite, capsule intake and potential therapy changes or abnormalities had to be reported. Patients were mainly examined by one investigator twice, at the beginning of the study (day 1; E1) and at the end of the study (after six weeks; E2). Examinations included blood sampling for routine analysis and for the determination of fatty acid profiles in blood plasma, a physical examination, measurement of skinfold thickness with a caliper and completion of the EORTC-QLQ-C30- (QoL in cancer patients), PAN26 (QoL in pancreatic cancer patients)-, and a nutrition questionnaire. The EORTC-QLQ-C30-questionnaire consists of 30 questions, which allow the determination of the following parameters: global health, functional (physical capacity and physical capacity concerning family and job, hobbies and emotions) and symptoms (pain, fatigue, nausea, diarrhea, appetite loss, obstipation, breathlessness, financial impact, sleeping disorders). The PAN26 questionnaire comprises of 26 questions assessing the parameters: pain, dietary changes, altered bowel habit, jaundice, emotional problems related to pancreatic cancer, cachexia, dry mouth, indigestion, flatulence and taste changes.

### Blood sampling and routine analysis

Blood samples were collected as EDTA blood (3 × S-Monovette® 9 ml with 1.6 mg EDTA/ml blood, Sarstedt, Nümbrecht, Germany) and centrifuged for 10 min at 2.000 rpm (805×g) at room temperature. The resulting plasmas were stored in aliquots of 500 μl at −80 °C until analysis. Routine blood parameters were measured by the clinical chemistry routine laboratory of the Tumor Biology Center Freiburg (CRP, albumin, thrombocytes, leukocytes, lipids (LDL, HDL, VLDL, total cholesterol, triglycerides)and liver enzymes (GOT, GPT, CHE).

### Evaluation of nutritional status

Patients were asked to report their daily body weight determined by the same body balance. Skinfold thickness was measured following the method of Durnin and Womersley [31] with a caliper at three body points: the upper arm, the back and on the iliac crest. Percentage of body fat was determined independently of water accumulation (edema or ascites).

### Fatty acid profile of blood plasma

Fatty acid (FA) analysis of the polar lipids (phospholipids) and non-polar lipids in patients plasma was performed as described by Taylor et al., 2010 [8]. In brief, polar and non-polar lipids were extracted with chloroform/methanol, followed by the separation of phospholipids and non-polar lipids using solid phase extraction with acetone. The FA-amounts and –compositions of both groups of lipids were measured by FA-methylation and subsequent gas chromatography (GC). 

### Statistics

Based on experience from the previous Phosfood study by Taylor et al. [8], sample size calculation resulted in16 patients and four drop outs per group, hence together 20 patients per group and 40 patients all in all. Because of the unexpected high number of dropouts, the size of the groups was raised to 60, altogether. Dropouts during the intervention were not included in the analysis, resulting in a per-protocol-analysis. Statistical analysis including t-test, Mann-Whitney-U-test, Wilcoxon-test, Pearson-correlation and Spearman-rang-correlation were performed with SigmaStat 3.1 (Systat Software Inc., USA, 2004), Mystat 12.02.00 (student version, Systat) and OriginPro 8 SR0.

## Results

### Study cohort

**Fig. 1:**
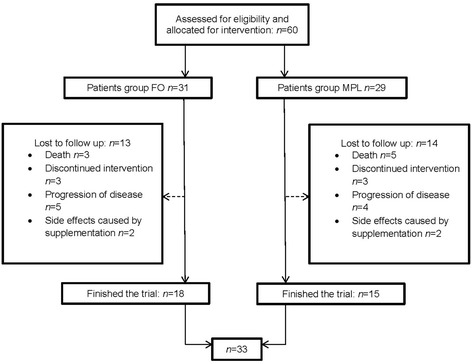
Patient enrollment: Flow chart of the Phosfood II dietary intervention 60 patients were randomized and allocated to the FO (*n* = 31) or MPL (*n* = 29) group. After six weeks of dietary intervention, 18 FO patients and 15 MPL patients could be analyzed (*n* = 33). FO = fishoil, MPL = marine phospholipids

Sixty patients were randomized and either assigned to the FO (*n* = 31) or MPL (*n* = 29) group. Thirteen out of 31 patients of the FO group and 14 out of 29 patients of the MPL group could not finish the study because of non-compliance, progressive disease, gastrointestinal side effects or death (Fig. 1). These patients were excluded from further analysis, since no data was available for an intention-to-treat analysis. Eighteen patients of the FO group and 15 patients of the MPL group completed the study and were examined for a second time. In both groups 12 patients received chemotherapy and one was treated with radiation, respectively. The remaining patients were on supportive or alternative therapies. Among the 18 patients of the FO group, there were 15 patients with a palliative therapy approach. Only three patients were treated with curative intention. In the MPL group, six patients had a curative therapy regime and nine patients had palliative treatment. Patients of the MPL group had a higher mean body weight six weeks before study start as well as at the beginning of the study, compared to patients in the FO group. Their mean BMI was also higher with 23.1 kg/m^2^ contrary to 21.3 kg/m^2^ (*p* = 0.02) (see Table 1 **).** Besides that, there were no significant differences between both groups.

**Table 1:** Baseline characteristics of the study population (*n* = 33)

Parameter	FO	MPL
Men/Women	7/11	9/6
Age (years)	70.3 ± 8.24	71.3 ± 7.51
BW 6 weeks before E1 (kg)*	62.9 ± 6.54	71.4 ± 15.3
BMI E1 (kg/m^2^)*	21.3 ± 1.73	23.7 ± 4.10
BW E1 (kg)	58.7 ± 4.93	67.6 ± 13.8
MM E1 (% BW)	21.5 ± 2.04	20.2 ± 2.98
FM E1 (% BW)*	25.2 ± 3.35	23.9 ± 2.74
BW without MM and FM (=body water) E1 (%)*	53.3 ± 2.80	55.5 ± 8.53

*non- normally distributed, *FO* fishoil, *MPL* marine phospholipids, *BW* body weight, *MM* muscle mass, *FM* fat mass, *E1* examination 1

### Appetite/meal portions

**Fig. 2:**
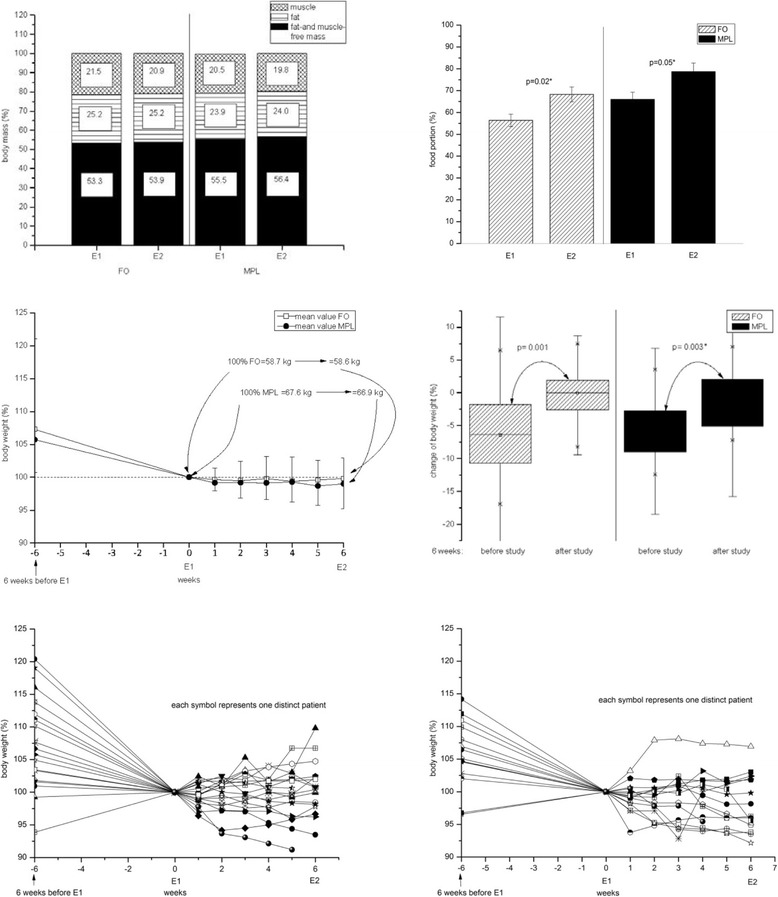
Body composition, food portions and body weight change. In both groups body composition remained nearly the same. Food portions increased in both groups. FO and MPL patients could stabilize their body weight after six weeks of dietary intervention compared to six weeks before intervention (left side FO. right side MPL). E1, E2 = examination1, 2, FO = fishoil. MPL = marine phospholipids, EPA = eicosapentaenoic acid

Both groups of patients experienced stabilization of appetite during the intervention. In accordance to that, meal portions increased significantly in both intervention groups (FO group (*p* = 0.02) and MPL-group (*p* = 0.05)) (see Fig. 2).

### Body weight

After six weeks of either FO or MPL intervention, both groups took advantage of their respective dietary supplementation, which became apparent in significant weight stabilization in comparison to the weight loss before the study (FO *p* = 0.001), MPL (*p* = 0.003), see Fig. 2). Body weight (BW) changes for all patients who finished the study are shown in fig. 2 (lower part) as dotted lines. Nine out of 18 patients (50%) of the FO group gained BW during the six week intervention. In the MPL group seven out of 15 patients (47%) gained weight. In both figures, the dotted lines show BW gain of two patients each in both groups six weeks before the study, which could be explained by water accumulation (edema and/or ascites). Taking this into account, these patients lost weight since their cancer diagnosis. As there was no overall significant increase in body water, BW gain seen in some of the patients can be explained by an increase in fat mass (FM) or/and muscle mass (MM). Considering the whole body constitution, there was no significant change in FM, MM and body water. With respect to the BMI, the MPL group started with a more advantageous body constitution than the FO group as the result of the randomization. None of the patients in the FO group had a BMI of 25 or higher. In contrast, six patients of the MPL group had a BMI of at least 25, which can be regarded as the upper limit of normal weight for healthy people, and which served as a reference value to the study. After the intervention, there was no significant statistical difference of BMI in both groups.

### Fatty acid composition

**Fig. 3:**
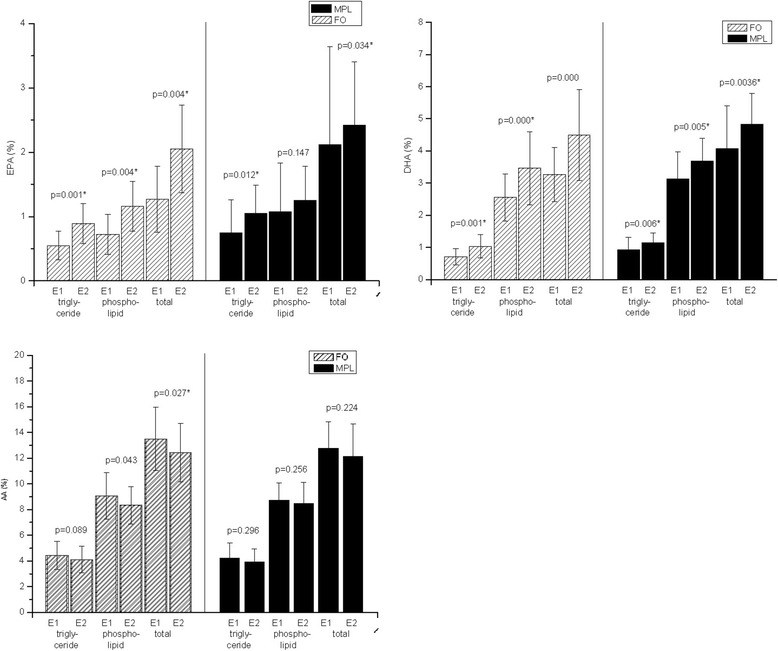
Fatty acid change. In both groups AA decreased after dietary intervention. The decrease was significant in the FO group. EPA and DHA increased in both groups significantly. Appetite correlated positively with total EPA at the second examination (left side FO, right side MPL)

During six weeks of either MPL or FO intake, most fatty acid profiles of both groups changed significantly. The average percentage of the anti-inflammatory docosahexaenoic acid (DHA) increased significantly in both, the plasma phospholipids as well as the the plasma triglycerides (FO: p = <0.01, *p* = 0.000; MPL: *p* = 0.005, *p* = 0.003; see Fig. 3, Table 2). The average percentage of the anti-inflammatory eicosapentaenoic acid (EPA) increased significantly in the plasma phospholipids and plasma triglycerides of the FO group (*p* = 0.002, *p* = 0.001; see Fig. 3 and Table 2). In contrast, in the MPL group, the average percentage of EPA only increased significantly in the plasma triglycerides (*p* = 0.01; see Fig. 3 and Table 2). The average percentage of the pro-inflammatory arachidonic acid (AA) decreased significantly in the phospholipid fraction of plasma in the FO group (*p* = 0.05; see Fig. 3 and Table 2), while no significant change was observed in the MPL group. Changes in fatty acid profiles also became apparent by comparing the n6/n3 ratios**.** Interestingly, the increase of EPA correlated positively with the improvement of appetite in the FO group (see Fig.3), in the MPL group there was no significant correlation observed.

**Table 2:** Mean plasma levels of n-3 and n-6 fatty acids in the FO group (*n* = 18) and MPL group (*n* = 15) at the beginning of the study (E1) and after 6 weeks (E2). All values are given in % of total fatty acids)

Fatty acid (mean)	E1	E2	*P*-value	change
EPA triglyceride				
FO	0.55 ± 0.22	0.89 ± 0.31	0.001**	+62%
MPL	0.76 ± 0.51	1.05 ± 0.44	0.01**	+38%
EPA phospholipid*				
FO	0.72 ± 0.31	1.16 ± 0.39	0.002**	+61%
MPL	1.08 ± 0.75	1.25 ± 0.53	0.20	+16%
EPA total*				
FO	1.27 ± 0.51	2.05 ± 0.68	0.001**	+61%
MPL	1.83 ± 1.23	2.31 ± 0.91	0.034**	+55%
DHA triglyceride*				
FO	0.71 ± 0.24	1.03 ± 0.36	<0.001**	+23%
MPL	0.94 ± 0.39	1.14 ± 0.30	0.003**	+14%
DHA phospholipid				
FO	2.55 ± 0.73	3.46 ± 1.14	0.000**	+36%
MPL	3.14 ± 0.83	3.69 ± 0.70	0.005**	+18%
DHA total				
FO	3.26 ± 0.84	4.49 ± 1.41	0.000**	+38%
MPL	4.07 ± 1.17	4.83 ± 0.96	0.004**	+19%
AA triglyceride				
FO	4.43 ± 1.08	4.12 ± 1.02	0.09	-7%
MPL	4.24 ± 1.15	3.94 ± 0.99	0.296	-7%
AA phospholipid*				
FO	9.07 ± 1.82	8.34 ± 1.45	0.05**	-8%
MPL	8.71 ± 1.37	8.51 ± 1.60	0.256	−2%
AA total				
FO	13.5 ± 2.47	12.5 ± 2.27	0.027**	-7%
MPL	13.0 ± 2.02	12.4 ± 2.37	0.22	−5%
n:6/n:3 (total)				
FO	3.13 ± 0.82	2.17 ± 1.11	<0.001**	-31%
MPL	2.44 ± 0.78	1.82 ± 0.49	0.000**	−25%

*non- normally distributed, *EPA* eicosapentaenoic acid, *DHA* docosahexaenoic acid, *AA* arachidonic acid

### Routine laboratory parameters

The measured parameters and their change after 6 weeks of n-3-FA intake in both intervention groups can be seen in Table 3 **.** Routine blood parameters only show significant changes in the FO group. HDL increased significantly from 42 mg/dl to 52 mg/dl (*p* = 0.002) in the FO group, the increase in the MPL group was not significant. The measurements at the beginning of the study showed that seven out of 18 patients of the FO group had a lower HDL level than recommended by the German Lipid-Liga (men and women: > 40 mg/dl, www.lipid-liga.de). In the MPL group there were only three out of 15 patients with a low HDL value. After six weeks patients in both, the FO and MPL group, who started with a low HDL level, experienced a significant increase of HDL during the intervention. On the contrary, patients who started with a high HDL did not experience a significant increase of HDL values after the intervention. Aside from lipid profile, there were also significant changes of GOT (*p* = 0.03) and thrombocytes (*p* = 0.01) in the FO group, but not in the MPL group.

**Table 3:** Basic blood parameters in the FO group (*n* = 18) and MPL group (*n* = 15) at the beginning of the study (E1) and after 6 weeks (E2)

Blood parameter (mean)	E1	E2	*P*-value	change
CRP (mg/dl)*				
FO	33.7 ± 65.8	15.6 ± 21.3	0.98	-54%
MPL	30.6 ± 68.9	10.4 ± 11.5	0.95	−66%
Albumin (g/dl)				
FO	3.73 ± 0.64	3.76 ± 0.63	0.76	+0,8%
MPL	3.69 ± 0.68	3.89 ± 0.55	0.31	+5,4%
Leukocytes (n/nl)*				
FO	7.78 ± 4.3	7.46 ± 4.93	0.59	-4%
MPL	7.88 ± 4.90	6.68 ± 2.09	0.40	−15%
Thrombocytes (n/nl)				
FO	296.3 ± 151.0	244.1 ± 121.1	0.01**	-18%
MPL	363.5 ± 254.6	270.9 ± 113.1	0.12	−25%
Triglycerides (mg/dl)				
FO	112.1 ± 45.2	99.3 ± 45.1	0.33	-11%
MPL	130.1 ± 78.0	116.6 ± 41.9	0.64	−10%
Cholesterol (mg/dl)				
FO	159.6 ± 47.0	161.8 ± 47.0	0.78	+1%
MPL	170.5 ± 42.7	179.2 ± 35.7	0.31	+5%
LDL (mg/dl)				
FO	90.8 ± 34.4	86.9 ± 39.4	0.54	-4%
MPL	99.7 ± 33.9	109.8 ± 33.6	0.11	+10%
HDL (mg/dl)*				
FO	51.3 ± 24.8	62.6 ± 28.7	0.00**	+22%
MPL	48.1 ± 16.4	50.4 ± 8.64	0.48	+5%
VLDL (mg/dl)*				
FO	20.4 ± 13.5	18.0 ± 9.4	0.29	-12%
MPL	23.9 ± 16.6	21.6 ± 10.9	0.74	-10%
GOT (U/l)*				
FO	39.3 ± 35.6	42.6 ± 32.3	0.03**	+8%
MPL	26.3 ± 7.41	29.4 ± 7.99	0.14	+12%
GPT (U/l)*				
FO	41.4 ± 44.9	48.4 ± 48.4	0.18	+17%
MPL	25.7 ± 8.99	36.0 ± 27.5	0.12	+40%
CHE (U/l)*				
FO	5558.8 ± 1880.3	5788.9 ± 1780.3	0.30	+4%
MPL	5388.9 ± 1630.8	5817.5 ± 1282.2	0.18	+8%
Ratio LDL/HDL				
FO	2.05 ± 0.90	1.63 ± 0.91	0.01**	-20%
MPL	2.6 ± 2.4	2.3 ± 1.04	0.60	-12%

E1, E2 = examination1, 2, * non-normally distributed, ** statistically significant

### Quality of life

In both groups there were no significant changes in QoL after six weeks of n-3 FA supplementation, which was measured with the EORTC-QLQ-C30-questionnaire, but slight positive changes in all the major parameters “physical”, “role”, “social”, “pain”, “appetite loss” and “global health” were observed. Using the PAN26 module, which was especially designed for pancreatic cancer patients, the parameter “hepatic”, decreased significantly in the MPL-group. “Hepatic” represents involvement of hepatic dysfunctions in the context of pancreatic adenocarcinoma.

**Fig. 4:**
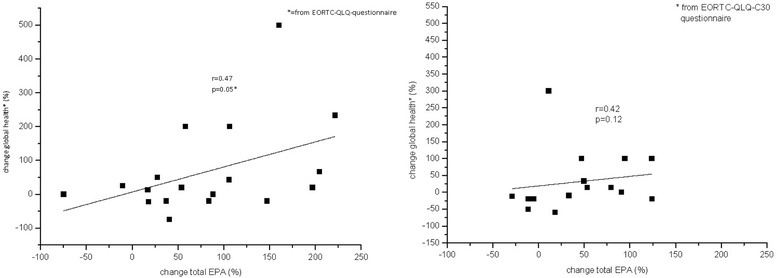
Correlation between the parameter “global health” and the total change of EPA. In the FO group, the change of total EPA correlated positively (*p* = 0.05) with the parameter “global health” of the EORTC-QLQ-C30 questionnaire, which stands for quality of life (QoL). In the MPL group, correlation was not significant (*p* = 0.12), (left side FO, right side MPL)

In addition, the observed increase of EPA in plasma correlated with the “global health” in the FO group (*p* = 0.05, *r* = 0.47, see Fig. 4). In the MPL group, no significant correlation was observed (*p* = 0.12, *r* = 0.42). There were no significant correlations between QoL and AA or DHA in both groups.

### Food questionnaire

Concerning food intake, there were no significant effects of both n-3-FA-formulations. At the beginning of the study (first examination) the n6/n3 ratio in the blood lipids correlated significantly with the reported consumption of fish (FO: *r* = −0.57, *p* = 0.02; MPL: *r* = 0.57, *p* = 0.03). Also, a significant correlation between EPA and fish intake at the first examination was observed in the MPL group (*r* = 0.49, *p* = 0.06).

### Compliance

In both groups the n-3 FA supplementation was highly accepted. In the FO group 94.8% and in the MPL group 97.6% of the patients took the supplement three times a day. Four patients of the FO group reported experiencing pyrosis, “fishy” regurgitation, loss of appetite, diarrhea and increased bowel movement. In the MPL group only one patient complained about diarrhea in the last week of the study. Additionally, at the end of the study, patients were asked if they would be willing to continue the n-3 FA supplementation. Seven out of 18 patients (38.9%) of the FO group argued for continuation of n-3-FA intake after the study. In the MPL group eleven out of 15 patients (73.3%) were willing to continue their supplementation. In some cases, patients reported taking further medications along with the study supplementation, which led them rather to stop taking the n-3-FA formulations after the end of the study.

## Discussion

One possibility to treat cancer cachexia is the oral application of anti-inflammatory n-3-FA. Usually, n-3-FA are given as triglycerides (fish oil, FO) and, according to a meta-analysis, the minimal effective dose is >1.5 g n-3-FA/day (EPA and DHA) [12]. This meta-analysis included 17 clinical trials reporting on the effects of n-3-FA for treating cachexia. Further studies showing positive effects of n-3-FA given as FO at even higher doses have been published [14–16, 20–22, 32–36].

Taylor et al. [8] investigated the effects of n-3-FA containing marine phospholipids (MPL) on cachectic cancer patients and found a weight stabilization effect at a five-fold lower dose (300 mg n-3-FA/day), showing at the same time a high tolerability. Ramprasath found similar results with another n-3-FA containing phospholipid formulation called krill oil. Based on these findings and in order to validate the effects of low dose n-3-FA containing phospholipids, we directly compared the effects of low dose FO and MPL carrying the same n-3-FA-composition on cachectic pancreatic cancer patients over six weeks.

Surprisingly, in both treatment arms the very low dosage of n-3-FA of 300 mg/day led to an impressive and similar median weight stabilization. While this effect was expected for the MPL group due to the study of Taylor (MPL) and Ramprasath (krill oil), the effect was unexpected for the group taking FO, due to the evidence, showing, that n-3-FA given as triglycerides will only be effective at a dose of >1.5 g/day. One possible reason for the weight stabilization effect in the FO group might be due to the addition of medium chain triglycerides (MCT, approximately 200 mg per capsule) to the FO formulation. MCT had to be included into the FO supplementation to guarantee the same capsule volume as the ones of the MPL supplementation. This was mandatory to achieve MPL and FO capsules with the same appearance and thus guarantee blinding. It was assumed that MCT would have no effect on body weight at the low dose of 200 mg/capsule. However, some studies have found body weight effects of high doses of MCT, as shown by Fearon et al. [22] In that study, MCT (given as placebo) was compared to the intake of 2 g and 4 g of EPA per day. The exact amount of MCT was not mentioned in his publication, but it can be assumed, that it was also 2 and 4 g,. Furthermore, MCT have often been successfully applied to treat weight loss in several studies not focusing on the effects of n-3-FA [37–39]. This seems plausible because of their better water solubility compared to long chain triglycerides and their faster intestinal absorption. MCT are an important element of diets for patients suffering from pancreatic insufficiency, cystic fibrosis as well as short barrel syndrome, as they serve for rapid energy generation [40–44]. The mentioned effects have been observed at much higher MCT doses (2.8 g MCT per day, Bounous et al. [43]), than administered in the current study.

The similar effects of low dose MPL and FO on the patient’s weight are not a random or placebo effect, which can be shown by the similar increase of n-3-FA in plasma triglycerides and phospholipids in both study groups. This allows us to assume, that the observed low dose MCT-effect is not due to the extra calories, but possibly due to a faster resorption of the n-3-FA induced by MCT in the FO group. One possible mechanism might be a better (or faster) availability of energy for mucosa cells, accelerating thereby the uptake of n-3-FA into chylomicrons. In contrast, the similar weight stabilization effect of MPL (without MCT) might be explained by a faster digestion and subsequent resorption of free n-3-FA or n-3-FA-carrying lyso-phospholipids, due to the expected rapid formation of a lipid emulsion of the MPL-contents in the gastric fluids. This emulsion consists most probably of rather small lipid particles, including also nanoparticles. The reason for that might be the fact that the ratio of the ‘emulsifier’ in the MPL formulation – the phospholipids – is much higher (~35%) than the ratio of phospholipids in commercial lipid emulsions for parenteral nutrition, which is typically about 3%. In MPL the ratio of phospholipids to triglycerides is more than ten times higher, which probably results in the self-assembly of very small lipid droplets. Those nanoparticles can easily be reached and hydrolyzed by lipolytic enzymes like pancreatic phospholipase A2 and pancreatic lipase [8].

The formation of an emulsion instead of forming larger lipid droplets in the gastric fluids also explains the better tolerability and fewer gastrointestinal side effects of MPL when compared to FO. This was also observed by Bruera et al. [20] and Burns et al. [23] As shown in that study, even when using a very low dose of FO, the tolerability of MPL was better than that of FO. We could observe only few side effects and a very high compliance in the MPL group. Patients were highly satisfied with this supplement and about 97.6% of the MPL group declared their wish to continue capsule intake after the end of the study.

Regarding body composition, the patients in the MPL group did not gain weight through accumulation of water, but by an increase of protein. For example, the female patient who had the lowest BMI at the beginning of the study (18.4 kg/m^2^) and who gained the most weight of all patients, increased her protein ratio by 10%. This corresponded to 84% of her weight gain, leading to a final BMI of 19.7 kg/m^2^. On the contrary another patient of the MPL group, who had the highest BMI at the beginning of the study (35.9 kg/m^2^) lost further 7 kg of body weight, which was almost completely water (final BMI: 33.3 kg/m2). On the other hand, patients in the FO group gained body water and lost protein, which was confirmed by skinfold thickness measurements. Further studies are necessary to confirm and explain these observations.

In terms of appetite, there was no statistical significant increase of appetite in both groups. This favors the hypothesis, that the observed weight stabilization was mainly caused by the suppression of inflammatory processes by n-3-FA and not due to a higher food intake. As the increase of EPA correlated positively with the improvement of appetite in the FO group, a possible appetite-stimulating effect of EPA can be discussed. However, since this effect could not be seen in the MPL group, this possibility remains unclear.

QoL was measured with two questionnaires (EORTC-QLQ-C30 and module PAN26). The analyzed parameters did not change significantly in both patient groups. Never the less, despite not significant, all the parameters have changed positively in both groups, assuming that there might be a positive effect of n-3-FA on QoL. These not significant improvements of QoL are in agreement with the findings of Taylor et al. [8] That there indeed might be an effect of n-3-FA on QoL is illustrated by the finding of a significant positive correlation between the general QoL-parameter “global health” and the increase of EPA in blood plasma after taking FO. Against our working hypothesis of a stronger effect of MPL on quality of life, when compared to FO, FO supplementation also results in an increase of the parameter “global health”. A possible reason for that could be the above discussed addition of MCT, which caused a similar increase of FO-bound n-3-FA in the blood plasma compared to MPL. Similar results were reported by Fearon et al. [22], who reported an improvement of “physical function” (as a component of “global health”) in patients taking FO supplementation. In a further study of Fearon et al. [21] with cachectic pancreatic carcinoma patients, the researchers found a significant positive correlation between FO consumption and QoL, as well as between QoL and gain of body weight. Furthermore, the parameter “hepatic”, measured by the PAN26 module, slightly decreased in the MPL group, which indicated less gastrointestinal dysfunctions. This might be explained by the liver protecting effect of the applied phospholipids: Gundermann et al. [45] summarized the beneficial effects of essential phospholipids from soybean in liver diseases. In his review he reported positive effects of phospholipids on inflammation, fibrosis and intoxication of the liver without relevant side effects [45]. If the effects of MPL on liver function are based on the phospholipids themselves or on a combination of phospholipids plus anti-inflammatory fatty acids, is not clear. Although the formulation used in this study is based on marine phospholipids, the active component remains the same, explaining the positive change of the parameter “hepatic” in the MPL group of this study.

## Conclusion

N-3-fatty acids, given as marine phospholipids or as fish oil in combination with medium chain triglycerides, are highly accepted and cause a significant weight stabilization effect after a period of six weeks at a very low dose of 300 mg n-3-FA/day in cachectic pancreatic carcinoma patients. In terms of weight stabilization, appetite and quality of life, effects of FO were similar to the effects of MPL, probably due to unexplained effects of low dosed MCT. However, MPL was better tolerated and had a higher acceptance in the study group.

Reflecting the higher compliance to MPL and the unexpected weight stabilizing effect of FO (with low dose of MCT), the question of an even more effective n-3-fatty acid formulation composed of MPL and MCT arises. Further studies are necessary to investigate the effects of MPL in a larger study population as well as to investigate the actual weight stabilizing mechanism of MCT.

## Abbreviations

AA: Arachidonic acid
EPA: Eicosapentaenoic acid
DHA: Docosahexaenoic acid
MPL: Marine phospholipids
BMI: Body mass index
BW: Body weight
CRP: C reactive protein
EORTC: European Organization for Research and Treatment of Cancer
PUFA: Polyunsaturated fatty acid
TG: Triglyceride
